# Resident Physician Experiences With and Responses to Biased Patients

**DOI:** 10.1001/jamanetworkopen.2020.21769

**Published:** 2020-11-23

**Authors:** Shalila S. de Bourmont, Arun Burra, Sarah S. Nouri, Neveen El-Farra, Dinushika Mohottige, Caroline Sloan, Sarah Schaeffer, Jodi Friedman, Alicia Fernandez

**Affiliations:** 1 San Francisco School of Medicine, University of California, San Francisco, San Francisco; 2 Department of Medicine, University of California, San Francisco, San Francisco; 3 Department of Medicine, University of California, Los Angeles, Los Angeles; 4 Division of Nephrology, Department of Medicine, Duke University School of Medicine, Durham, North Carolina; 5 Department of Medicine, Duke University School of Medicine, Durham, North Carolina

## Abstract

**Importance:**

Biased patient behavior negatively impacts resident well-being. Data on the prevalence and frequency of these encounters are lacking and are needed to guide the creation of institutional trainings and policies to support trainees.

**Objective:**

To evaluate the frequency of resident experiences with and responses to a range of biased patient behaviors.

**Design, Setting, and Participants:**

A retrospective survey was sent via email to 331 second- and third-year internal medicine residents from 3 academic medical centers in California and North Carolina. First-year residents were excluded owing to their limited interactions with patients at the time of participant recruitment. Data were collected from August 21 to November 25, 2019.

**Main Outcomes and Measures:**

Descriptive statistics were used to report the frequency of experience of various types of biased patient behavior, residents’ responses, the factors impeding residents’ responses, and residents’ experiences and beliefs regarding training and policies.

**Results:**

Overall, 232 of 331 residents (70%) participated; 116 (50%) were women; 116 of 247 (47%) were White (participants had the option of selecting >1 race/ethnicity); and 23 (10%) identified as lesbian, gay, bisexual, transgender, or queer. The frequency of resident-reported experience of types of biased patient behaviors varied. The most common behaviors—belittling comments and assumption of nonphysician status—were reported to be experienced 1 or more times per week by 14% of residents (32 of 231) and 17% of residents (38 of 230), respectively. Women, Black or Latinx, and Asian residents reported experiencing biased behavior more frequently. Forty-five percent of Black or Latinx residents (17 of 38) encountered instances of explicit epithets or rejection of care. All 70 Asian residents reported experiencing inquiries into their ethnic origins. Most women residents (110 of 115 [96%]) experienced role questioning behaviors, and 87% (100 of 115) experienced sexual harassment. The need to prioritize clinical care and a sense of futility in responding were the most common factors (cited by 34% of residents [76 of 227] and 25% of residents [56 of 227], respectively) significantly impeding responses to biased behaviors. Eighty-five percent of residents (191 of 226) never reported incidents to their institution. Eighty-nine percent of residents (206 of 232) identified training and policies as necessary or very necessary.

**Conclusions and Relevance:**

This survey study suggests that biased patient behavior is experienced frequently by internal medicine residents. Non-White and women residents reported experiencing a disproportionate burden of these incidents. Residents’ responses rarely included institutional involvement. Residency programs and health care systems should prioritize training and policies to address biased patient behavior and support affected residents.

## Key Points

**Question:**

How often do resident physicians encounter incidents of biased patient behavior, and how do they respond?

**Findings:**

In a survey study of 232 internal medicine residents from 3 institutions, biased patient behavior ranging from belittling comments to refusal of care was experienced or witnessed by nearly all residents. Forty-five percent of Black/Latinx residents experienced epithets or refusal of care, and most women (87%) experienced sexual harassment; however, most residents (84%) did not report these encounters to their institutional leadership.

**Meaning:**

Given the high prevalence of biased patient behavior, residency programs that aim to foster inclusive training environments should implement resident and faculty training and create patient reporting mechanisms.

## Teaser

This survey study evaluates the frequency of resident physician experiences with and responses to a range of biased patient behaviors.

## Introduction

Patients who demean physicians on the basis of physicians’ social characteristics pose multiple clinical and ethical challenges.^1,2^ Biased patient behaviors arise when patients encounter physicians whose social identity (eg, race, ethnicity, or gender identity) is not compatible with their notion of a competent or appropriate health care professional. Biased patient behaviors can range from offensive quips to outright refusal of care^3^ and can exact a heavy psychological toll on physicians.^2,3,4,5,6,7,8,9^ It is possible that physicians’ encounters with biased patients have become more common in recent years: the health care workforce is increasingly diverse along multiple social dimensions, and the number of hate groups and hate crimes in the US has grown during the past 5 years, suggesting increased social polarization.^10^ Characterizing the nature and frequency of these occurrences may be particularly important for academic medical centers, as these incidents undermine learning for trainees and may result in delayed or suboptimal patient care.^3,4^

Data on the frequency of these occurrences among trainees—and even more so on physicians’ responses to them—are limited, as much of the literature consists of first-person physician accounts of interactions with racist patients or online surveys of practicing physicians.^5,6,7,11,12,13,14,15,16,17,18^ A recent national survey of general surgery residents reported that 43% of residents experienced discrimination on the basis of gender identity and 47% experienced racial/ethnic discrimination from patients or patients’ families.^17^ The same study also found that residents who experienced demeaning behavior were more likely to experience burnout and to have suicidal thoughts. In a qualitative study, internal medicine residents who encountered biased patients reported experiencing painful emotions including fear, self-doubt, exhaustion, and cynicism; residents who did not have these encounters reported moral distress and uncertainty about how to respond and support their colleagues when these situations arise.^3^

To create institutional policies to support and teach residents and faculty how to manage these challenging patient encounters, academic medical centers and medical educators need data on the frequency of specific types of biased patient behaviors as well as a better understanding of how resident experience may vary according to social characteristics, such as gender identity and race/ethnicity. In addition, it is important to know how residents commonly respond to these behaviors and what individual, patient-related, or institutional elements factor into their decisions on how to respond. We conducted a survey of physician residents at 3 large, academic internal medicine residency programs to evaluate the frequency of resident experiences with and responses to a range of biased patient behaviors.

## Methods

### Study Setting and Participants

We administered an electronic survey to second- and third-year internal medicine residents at 3 academic medical centers in California and North Carolina (University of California, San Francisco; University of California, Los Angeles; and Duke University). We collected data from August 21 to November 25, 2019. We excluded first-year residents owing to their limited interactions with patients at the time of participant recruitment. The institutional review boards at each site approved the study, and all participants provided written informed consent. This study followed the American Association for Public Opinion Research (AAPOR) reporting guideline.^19^

### Survey Development

We developed a survey with both multiple choice and open-ended questions based on prior qualitative research exploring the range of demeaning behaviors experienced by physicians and trainees, types of responses to such behavior, and barriers to responding.^3^ The survey was iteratively revised by the study team. We conducted cognitive testing with residents and faculty from the University of California, San Francisco Department of Medicine to assess the overall coherence, balance, and clarity of the survey. The full survey is available in the eAppendix in the Supplement.

### Survey Measures

#### Experiences With Biased Patient Behaviors

We asked participants to indicate how frequently they had directly experienced the following demeaning behaviors from patients: (1) belittling or demeaning stereotypes (belittling comments, inquiries into racial/ethnic origins, generalizations about social identity, confusing physician with team member of the same race/ethnicity, and nonverbal disrespect), (2) role questioning (credential questioning, assumption of nonphysician status, and addressing less experienced physician or student rather than the participant), (3) explicit epithets or rejection of care (epithets, refusal of care, and request to change physician), and (4) sexual harassment (verbal or nonverbal sexual advances or sexual harassment). Participants were provided the option to describe other demeaning behaviors not defined above. These behaviors are hereafter referred to as “biased patient behaviors.”

Participants were asked to indicate how frequently patients targeted their own social identities (race/ethnicity or national identity, gender identity or expression, Muslim faith, non-Muslim faith, sexual orientation, and disability status) and how frequently they witnessed biased patient behavior targeting other physicians’ social identities. Response options were classified as never, sometimes (once to a few times per year), often (once to multiple times per month), or very often (at least once per week).

#### Residents’ Responses to and Training on Biased Patient Behaviors

We asked how residents respond to these biased patient behaviors (including types and frequencies of responses, barriers to responding, and confidence in responding), residents’ prior experience with training on how to respond to these behaviors, and the degree to which residents believe that training and institutional policies for guiding responses are necessary. We asked about specific responses (1-on-1 limit setting, debriefing with friends or family, debriefing with a team member, creating a team response plan, reporting to attending physician or chief resident, reporting to the institution, switching the patient to another team member, and not addressing the incident). In addition, we asked about the negative impact (from “no impact” to “significant impact”) of specific barriers on responding (prioritizing clinical care; feeling unsupported by the team, senior physicians, or institution; lack of knowledge or skills; perceived ineffectiveness or sense of futility in responding; and feeling emotionally overwhelmed). Participants could also describe other responses and barriers not defined above.

#### Demographic Characteristics

Participants were asked to report their clinical postgraduate year; gender identity; race/ethnicity; identification as lesbian, gay, bisexual, transgender, or queer (LGBTQ) or as another sexual or gender minority; and whether they were an immigrant to the US. Options were defined by the research team with an open field option for participants to describe demographics outside those listed. These demographic characteristics were collected to understand how residents’ experiences varied by social characteristics.

### Study Procedures

Participants were emailed a link to an anonymous survey via Qualtrics. Follow-up reminder emails were sent to nonrespondents as needed by study authors as well as chief residents at each institution. Participants who completed the survey were emailed a gift card for $10.00 USD to Amazon.

### Analysis

We conducted descriptive analyses of (1) the prevalence of residents’ experiences with biased patient behaviors overall that were stratified by resident race/ethnicity and gender identity, (2) the frequency of behaviors targeting specific resident identities, (3) the frequency of witnessed biased patient behaviors targeting other residents’ identities, and (4) the types and frequencies of residents’ responses to biased patient behaviors. Because of small sample sizes, we combined participants who identified as Black and/or Latinx into 1 category (Black + Latinx) for analysis. In reporting the frequency of different categories of behaviors stratified by race/ethnicity and gender identity, we present means of the component behaviors of those categories.

Two of us (A.B. and S.S.N.) independently reviewed all responses to open-ended questions about experiences with and responses to biased patient behavior and coded responses using grounded theory.^20^ The reviewers then met to reconcile discrepancies and define themes, which allowed identification of new categories of patient behavior and resident responses not defined in the survey.

## Results

### Participant Characteristics

Of 331 residents, 232 (70%) responded to the survey. The response rate (using AAPOR Response Rate Calculation 2) was 70% at each of the 3 participating programs.^19^ Residents were diverse in gender identity and race/ethnicity (Table 1). Residents identifying as LGBTQ or another gender or sexual minority made up 10% of participants (23 of 232); 16% of participants (36 of 232) identified as immigrants to the US. Respondents were representative of the racial/ethnic and gender identity demographics of their residency programs as a whole.

**Table 1.** Characteristics of Participants

Characteristic	Participants (N = 232)
Institution, No. (%)	
1	71 (31)
2	79 (34)
3	82 (35)
Gender identity, No. (%)	
Man	113 (49)
Woman	116 (50)
Other	2 (1)
Identify as LGBTQ or gender or sexual minority, No. (%)	23 (10)
Race/ethnicity, No./total No. (%)^a^	
White	116/247 (47)
Black or African American	13/247 (5)
Hispanic or Latinx	27/247 (11)
Asian	72/247 (29)
Native Hawaiian or Pacific Islander	3/247 (1)
Native American or Alaska Native	1/247 (0.4)
Other	15/247 (6)
Immigrant to the US, No. (%)	36 (16)
Year of residency, No. (%)	
PGY-2	131 (57)
PGY-3	101 (44)
Prior training on patient bias during residency, No. (%)	
None	44 (19)
<1 h	49 (21)
1-2 h	94 (41)
>2 h	45 (19)
Prior training on patient bias during medical school, No./total No. (%)	
None	65/230 (28)
<1 h	60/230 (26)
1-2 h	66/230 (29)
>2 h	39/230 (17)

Abbreviations: LGBTQ, lesbian, gay, bisexual, transgender, or queer; PGY, postgraduate year.

^a^ Participants had the option of selecting more than 1 race/ethnicity.

### Prevalence of Types of Biased Behaviors

Nearly all residents (228 of 232 [98%]) reported experiencing or witnessing biased behavior at least once in the past year. The frequency of specific biased patient behaviors varied (Table 2). A total of 14% of residents (32 of 231) experienced belittling comments at least once a week, 11% (25 of 230) experienced questioning of credentials or abilities, and 17% (38 of 230) experienced assumption of nonphysician status occurring at least once a week. Behaviors reported by one-third of participants as occurring at least once per month included belittling comments (87 of 230 [38%]), assertive inquiries into racial/ethnic origins (75 of 231 [33%]), generalizations about social identity (70 of 231 [30%]), and credential or ability questioning (77 of 230 [34%]). In contrast, epithets, refusal of care, and requests to change physicians were less common yet were experienced at least 1 to 3 times per year by 40% of residents (91 of 230), 30% of residents (69 of 230), and 27% of residents (61 of 229), respectively. Sexual harassment was also common and experienced at least 1 time per year by 60% of participants (138 of 230).

**Table 2.** Prevalence of Direct Experiences of Types of Biased Patient Behavior in the Last Year

Type of behavior	Respondents, No./total No. (%) (n = 231)^a^			
	Never	Sometimes	Often	Very often
Belittling or demeaning stereotypes^b^				
Belittling comments	47/230 (20)	64/230 (28)	87/230 (38)	32/230 (14)
Inquiries into racial/ethnic origins	77/231 (33)	68/231 (29)	75/231 (33)	11/231 (5)
Generalizations about social identity	41/231 (18)	105/231 (46)	70/231 (30)	15/231 (7)
Confusing team members of the same race/ethnicity	53/231 (23)	91/231 (39)	65/231 (28)	22/231 (10)
Nonverbal disrespect	95/230 (41)	111/230 (48)	22/230 (10)	2/230 (1)
Role questioning^b^				
Credential or ability questioning	37/230 (16)	91/230 (40)	77/230 (34)	25/230 (11)
Assumption of nonphysician status	77/230 (34)	48/230 (21)	67/230 (29)	38/230 (17)
Addressing intern or student because of social bias toward senior resident	98/228 (43)	69/228 (30)	49/228 (22)	12/228 (5)
Sexual harassment^b^	92/230 (40)	98/230 (43)	38/230 (17)	2/230 (1)
Explicit epithets or rejection of care^b^				
Epithets	139/230 (60)	79/230 (34)	11/230 (5)	1/230 (0.4)
Refusal of care	161/230 (70)	65/230 (28)	4/230 (2)	0
Request to change physicians	168/229 (73)	59/230 (26)	2/230 (1)	0

^a^ *Sometimes* was defined as 1 to 2 or a few times per year, *often* was defined as once or more than once per month, and *very often* was defined as once per week or more.

^b^ Because of missing data, the total No. was less than 231 and ranged between 228 and 230.

Experiences with biased patient behavior were more common for residents who identified as women, Black or Latinx, and Asian (Figure). Most residents identifying as women (100 of 115 [87%]), Latinx or Black (29 of 38 [76%]), and Asian (43 of 70 [61%]) reported experiences of sexual harassment within the last year compared with 32% of those identifying as men (36 of 113) and 54% of those identifying as White (59 of 109) (Figure). In addition, 96% of women residents (110 of 115) reported encountering role-questioning behaviors at least once within the past year compared with 42% of male residents (47 of 113) (Figure). All 70 residents identifying as Asian reported experiencing inquiries into ethnic origins, and 99% (69 of 70) reported being confused with team members of the same race/ethnicity at least once within the past year (eTable 1 in the Supplement). All 115 residents identifying as women reported experiencing assumptions of nonphysician status at least once within the past year (eTable 1 in the Supplement). Experiences of refusal of care and requests to change physicians were more common among residents identifying as Black or Latinx—45% (17 of 38) reported encountering these behaviors within the last year compared with 28% of White-identifying residents (31 of 109) (Figure).

**Figure.**
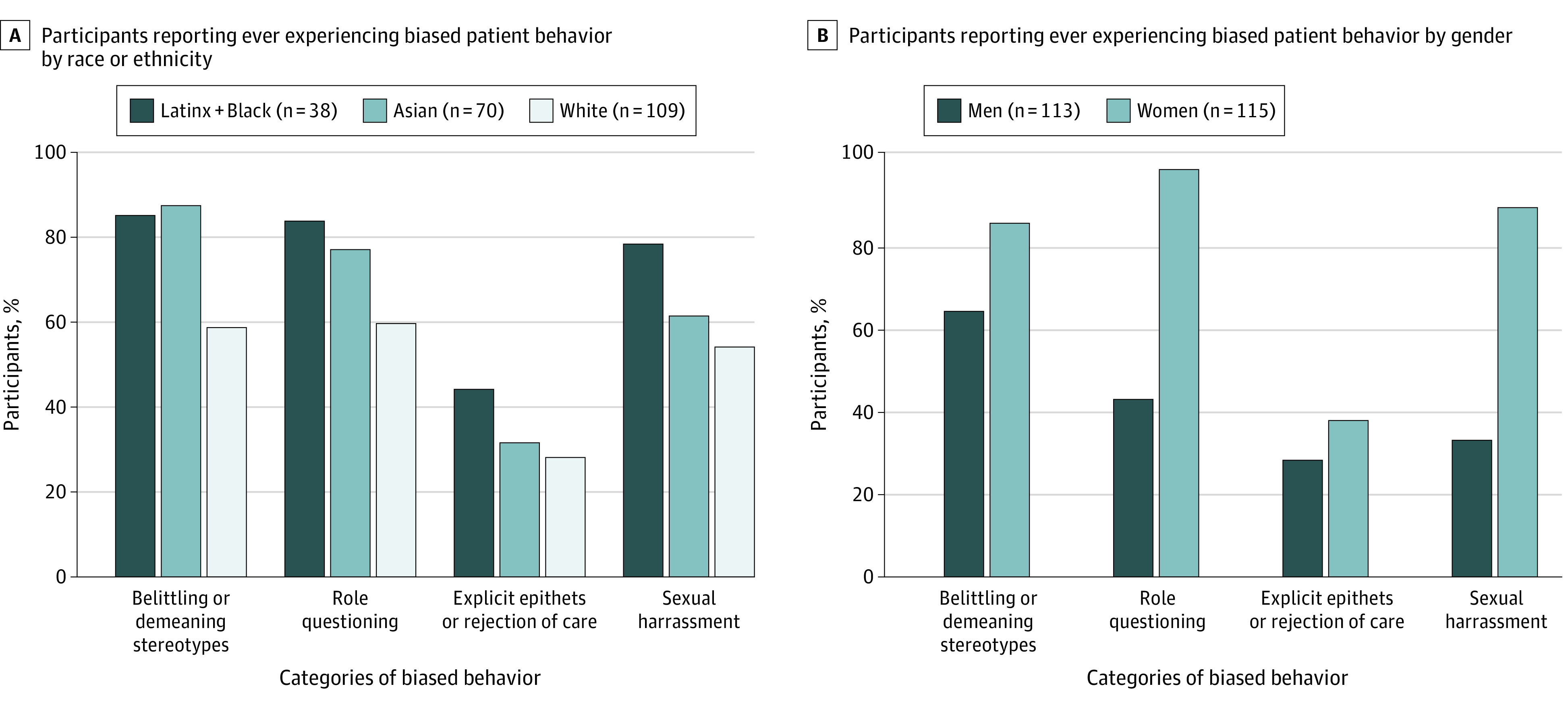
Percentage of Participants Reporting Ever Experiencing Biased Patient Behavior by Race/Ethnicity and Gender Identity

In general, physicians reported witnessing all types of biased behavior more frequently than they reported directly experiencing it (eTable 2 in the Supplement). Biased patient behavior directed toward a physician’s race/ethnicity or national identity and gender identity or expression were witnessed very commonly, with 89% of residents (205 of 230) and 74% of residents (169 of 230), respectively, reporting having witnessed at least one instance within the past year. Behavior directed toward a physician’s sexual orientation and Islamic faith were also common, as 42% of residents (95 of 227) and 40% of residents (91 of 228), respectively, reported witnessing that behavior at least once within the past year (eTable 2 in the Supplement). Finally, content analysis of written responses did not reveal any additional types of biased behavior.

### Responses and Barriers to Responses

In response to biased patient behavior, residents used strategies such as limit setting (67 of 227 [30%]), debriefing with friends or family (80 of 227 [35%]), and debriefing with team members (77 of 225 [34%]) most of the time or always (Table 3). Reporting biased patient behavior to the institution or switching the patient to another team member were uncommon: most residents reported never using those options (85% [191 of 226] and 78% [176 of 226], respectively). The factors with the greatest negative association with a resident’s ability to respond to biased behavior included the need to prioritize clinical care and a sense of futility in responding, with 34% of residents (76 of 227) and 25% of residents (56 of 227), respectively, identifying these factors as having significant impact (Table 4). Content analysis of written comments also revealed a few additional responses; most commonly, a witness in the room addressed biased behavior. Rarer responses included documenting biased behavior in the electronic medical record and withdrawing from the interaction with the offending patient as much as possible.

**Table 3.** Frequency of Responses Used to Address Biased Patient Behavior

Type of response	Respondents, No./total No. (%) (n = 227)			
	Never	Sometimes	About half the time	Frequently^a^
1-on-1 Limit setting	22/227 (10)	93/227 (41)	45/227 (20)	67/227 (30)
Debriefing				
With friends or family	36/227 (16)	77/227 (34)	34/227 (15)	80/227 (35)
With team members^b^	11/225 (5)	72/225 (32)	65/225 (29)	77/225 (34)
Creating team response plan^b^	104/226 (46)	79/226 (35)	23/226 (10)	20/226 (9)
Reporting to attending physician or chief resident	106/227 (47)	74/227 (33)	20/227 (9)	27/227 (12)
Reporting to institution^b^	191/226 (84)	29/226 (13)	2/226 (1)	4/226 (2)
Switching patient to another team member^b^	176/226 (78)	41/226 (18)	6/226 (3)	3/226 (1)
Not addressing the incident^b^	47/225 (21)	120/225 (53)	29/225 (13)	29/225 (13)

^a^ *Frequently* was defined from survey categories as occurring most of the time or always.

^b^ Because of missing data, the total No. was less than 227 and ranged between 225 and 226.

**Table 4.** Factors Impeding Residents From Responding to Biased Patient Behavior

Factor	Impact, No. (%) (n = 227)			
	None	Minimal	Some	Significant
Prioritizing the clinical care of the patient	16 (7)	45 (20)	90 (40)	76 (34)
Feeling unsupported by the team, senior physicians, or institution	84 (37)	88 (39)	50 (22)	5 (2)
Lack of knowledge or skills about how to properly respond	33 (15)	69 (30)	97 (43)	27 (12)
Perceived ineffectiveness of responding	27 (12)	41 (18)	94 (41)	56 (25)
Feeling emotionally overwhelmed	42 (19)	71 (31)	77 (34)	37 (16)

### Training and Policies

Only 32% of residents (73 of 231) were confident or very confident in responding to biased patients (eTable 3 in the Supplement). Most residents (206 of 232 [89%]) identified training for medical students, residents, and faculty on dealing with biased patients as necessary or very necessary; similar numbers believed institutional policies to be necessary or very necessary for addressing biased patient behavior (eTable 4 in the Supplement). With regard to training, 81% of residents (187 of 232) reported receiving 2 hours or less of training on caring for biased patients during residency (Table 1). Although 72% of residents (165 of 230) had prior training on patient bias in medical school, 74% (150 of 203) rated the content of their prior training as below adequate (eTable 5 in the Supplement).

## Discussion

In this survey study of second-year and third-year residents at 3 large, academic internal medicine residency programs, we found that nearly all residents reported witnessing or experiencing biased patient behavior within the past year. The frequency and extent of specific biased patient behaviors varied substantially, with belittling comments experienced on a monthly or weekly basis by more than half the residents and bias-based requests to change physicians experienced a few times a year by one-third of residents. Although ethnicity/race or national origin and gender identity or expression were the most commonly targeted sociodemographic characteristics, sexual harassment was particularly prevalent. Most residents do not report these encounters to institutional leadership, preferring instead to respond to incidences of patient bias on their own or debrief with friends, family, and team members.

To address the issue of biased patient behavior, interventions are needed at the institutional and interpersonal levels. The high prevalence of biased patient incidents may be largely unknown by residency program directors and academic medical center leaders as evidenced by the 80% of residents who stated that they never reporting biased patient behavior “up” within their institution. At the institutional level, low use of a formal structure to address and report incidents may indicate either a lack of formal reporting processes, a lack of knowledge of existing processes, or a reluctance to engage in these processes for fear of negative repercussions. This finding should be explored further and clear reporting mechanisms instituted, while recognizing that reporting systems succeed only when institutional culture encourages reporting without fear of retaliation. Specific policies on approaching patients who explicitly reject or refuse care based on sociodemographic bias requires a specialized approach to navigate the ethical and legal implications of such behaviors.^1^ On an interpersonal level, training on dealing with biased patients should be incorporated into resident and faculty development curricula. Most residents described prior training on biased patient behavior as below adequate, and only one-third felt confident in responding to biased patient behavior. Developing a deeper understanding of residents’ sense of futility in responding to bias will be necessary for effective development and implementation of trainings and policies. Well-facilitated team debriefing should be continued through allocated time and space where residents’ experiences can be acknowledged, validated, and addressed.^21^ Bystander trainings on supporting targeted colleagues should also be encouraged given how frequently residents reported witnessing biased behavior.

Our results are consistent with those of prior studies of harassment and discrimination of physicians that have found a disproportionate burden on those identifying as women and/or racial/ethnic minorities.^17,22,23,24,25,26,27,28^ We found that Latinx and Black residents experienced all types of biased behaviors more frequently than their White counterparts. More than 40% of Latinx and Black residents experienced the more extreme forms of patient bias—racial epithets, bias-based refusals of care, and bias-based requests to change physician—at least once in the last year. Academic medical centers need to communicate clear policies on physician refusal based on race/ethnicity to their patients and key medical educators, including attending physicians, chief residents, and other front-line supervisors.^1^

Asian residents reported the greatest prevalence of belittling and demeaning behaviors. All Asian respondents surveyed reported bias-based inquiries into their ethnic origins. Asian residents also experienced more role questioning than White residents. Sociologists have highlighted the “perpetual foreigner” status of Asian Americans, wherein Asian immigrants or their descendants are considered to be not fully US citizens or to be more closely tied to their country of origin than to the United States.^29^ Given the high prevalence of biased patient behaviors experienced by Asian residents—and the common misconception that Asians are a homogeneous, overrepresented group in medicine (in actuality, many Asian subgroups, such as Cambodian Americans, are underrepresented)^30^—our data highlight the need to consider the Asian American trainee experience when designing programs to address patient bias. Moreover, such support is particularly urgent given the increase in biased behavior and hate crimes toward Asian Americans in the time of coronavirus disease 2019 (COVID-19).^31^

Consistent with other reports,^32,33^ we noted a high prevalence of sexual harassment. Eighty-seven percent of female respondents reported experiencing verbal or nonverbal sexual harassment, meaning that this behavior may well be regarded as a norm within clinical practice. We also found that a common barrier to responding to biased patient behavior is a sense of futility, reflecting a culture that rarely holds abusers accountable. Harassment constitutes one of many gender identity–based inequities in medicine alongside gaps in salary, career advancement, and leadership that will require a concerted effort to address.^34^

Prior qualitative work has shown that individuals reporting multiple marginalized group identities often encounter uniquely complex, compounded discriminatory behavior.^3^ This concept of intersectionality is critical to understanding, validating, and addressing the multidimensional experiences faced by individuals who are members of multiple marginalized groups (eg, ability status, race/ethnicity, immigration status, nationality, religion, sex, gender identity or expression, and sexual orientation).^35,36^ Our ability to examine the frequency of biased patient behavior toward these individuals in our study was limited by small sample size. Larger studies are needed to measure the frequency and nature of bias that trainees belonging to multiple marginalized groups experience in the workplace.

### Limitations

Our study has several limitations. First, survey responses are inherently subject to recall bias. Second, as noted, our sample size was too small to analyze the impact of the intersection of identities or evaluate the experience of respondents with less common social identities, such as those who identified as gender nonbinary. Third, while we surveyed residents in 3 urban areas in 2 geographically distinct regions of the US, our findings may not reflect the experiences of residents in other parts of the country. Despite these limitations, our study is, to our knowledge, the first to evaluate the frequency of specific biased patient behaviors and physician responses to these behaviors. Prior studies have shown that asking about specific behaviors greatly increases reporting of harassment,^32^ suggesting that prior studies without this survey’s level of granularity may have underreported these behaviors.

## Conclusions

In this survey study, resident physicians’ reported that experiences with biased patient behavior were common, varied both in the type of behavior and the intended target, and were disproportionately prevalent for women, Latinx, Black, and Asian residents. Despite the diversification of the physician workforce and the steady work to decrease bias and discrimination within the workplace, there has been a lack of training and policies to address the social, emotional, and clinical challenges that biased patient behavior produces. Addressing biased patient behavior will require medical institutions to bring forth training, policies, and cultural change that support their trainees and workforce and promote an environment free from harassment and bias for physicians and other health care workers.

## Notes

Supplement. **eTable 1.** Percentage of Participants Reporting Ever Experiencing Biased Patient Behavior by Race/Ethnicity and Gender **eTable 2.** Percentage of Participants Ever Experiencing or Witnessing Biased Patient Behavior Targeted at Specific Social Characteristics in the Last Year **eTable 3.** Confidence in Responding to Biased Patient Behavior **eTable 4.** Beliefs Around Necessity of Policies and Training on Addressing Biased Patient Behavior **eTable 5.** Beliefs on Adequacy of Prior Training on Biased Patient Behavior in Terms of Content and Time **eAppendix.** Resident Experiences With Biased Patient Behavior Survey

## References

[1] Paul-Emile K, Smith AK, Lo B, Fernández A Dealing with racist patients. N Engl J Med. 2016;374(8):708-711. doi:10.1056/NEJMp1514939 26933847

[2] Reynolds KL, Cowden JD, Brosco JP, Lantos JD When a family requests a white doctor. Pediatrics. 2015;136(2):381-386. doi:10.1542/peds.2014-2092 26169433

[3] Wheeler M, de Bourmont S, Paul-Emile K, Physician and trainee experiences with patient bias. JAMA Intern Med. 2019;179(12):1678-1685. doi:10.1001/jamainternmed.2019.4122 31657839 PMC6820043

[4] Mustapha T, Ho Y, Andrews JS, Cullen MJ See no evil, hear no evil, stop no evil: institutional-level tracking to combat mistreatment of residents and fellows. J Grad Med Educ. 2019;11(5):601-605. doi:10.4300/JGME-D-19-00218.1 31636833 PMC6795325

[5] Watson S Credentials don’t shield doctors, nurses from bias. WebMD. Published October 18, 2017. Accessed January 17, 2019. https://www.webmd.com/a-to-z-guides/news/20171018/survey-patient-bias-toward-doctors-nurses

[6] Reddy S How doctors deal with racist patients. *Wall Street Journal* January 22, 2018. Accessed December 18, 2018. https://www.wsj.com/articles/how-doctors-deal-with-racist-patients-1516633710

[7] Okwerekwu JA What happened when I talked about what others ignore—racism in medicine. *STAT News* Published April 27, 2016. Accessed June 17, 2019. https://www.statnews.com/2016/04/27/racism-medicine-lessons/?s_campaign=medscape

[8] Nunez-Smith M, Curry LA, Bigby J, Berg D, Krumholz HM, Bradley EH Impact of race on the professional lives of physicians of African descent. Ann Intern Med. 2007;146(1):45-51. doi:10.7326/0003-4819-146-1-200701020-00008 17200221

[9] Sue DW, Capodilupo CM, Torino GC, Racial microaggressions in everyday life: implications for clinical practice. Am Psychol. 2007;62(4):271-286. doi:10.1037/0003-066X.62.4.271 17516773

[10] Southern Poverty Law Center Hate groups reach record high. Published February 19, 2019. Accessed October 28, 2019. https://www.splcenter.org/news/2019/02/19/hate-groups-reach-record-high

[11] Warshaw R. When the target of bias is the doctor. *AAMC News* Published May 9, 2017. Accessed April 19, 2019. https://news.aamc.org/patient-care/article/target-bias-doctor/

[12] Jain SH The racist patient. Ann Intern Med. 2013;158(8):632. doi:10.7326/0003-4819-158-8-201304160-00010 23588752

[13] Olayiwola JN Racism in medicine: shifting the power. Ann Fam Med. 2016;14(3):267-269. doi:10.1370/afm.1932 27184998 PMC4868566

[14] Astik G. I don’t want someone like YOU caring for me. *The Hospital Leader* Published January 17, 2019. Accessed June 17, 2019. https://thehospitalleader.org/i-dont-want-someone-like-you-caring-for-me/

[15] Youmans QR The N-word. Ann Intern Med. 2019;171(5):380-381. doi:10.7326/M19-1269 31476228

[16] Tedeschi B. 6 in 10 Doctors report abusive remarks from patients, and many get little help coping with the wounds. *STAT News* Published October 18, 2017. Accessed June 17, 2019. https://www.statnews.com/2017/10/18/patient-prejudice-wounds-doctors/?s_campaign=medscape

[17] Hu YY, Ellis RJ, Hewitt DB, Discrimination, abuse, harassment, and burnout in surgical residency training. N Engl J Med. 2019;381(18):1741-1752. doi:10.1056/NEJMsa1903759 31657887 PMC6907686

[18] vanIneveld CH, Cook DJ, Kane SL, King D; Internal Medicine Program Directors of Canada Discrimination and abuse in internal medicine residency. J Gen Intern Med. 1996;11(7):401-405. doi:10.1007/BF02600186 8842931

[19] The American Association for Public Opinion Research Standard Definitions: Final Dispositions of Case Codes and Outcome Rates for Surveys. 9th ed American Association for Public Opinion Research; 2016.

[20] Strauss A. Qualitative Analysis for Social Scientists. Cambridge University Press; 1987. doi:10.1017/CBO9780511557842

[21] Shankar M, Albert T, Yee N, Overland M Approaches for residents to address problematic patient behavior: before, during, and after the clinical encounter. J Grad Med Educ. 2019;11(4):371-374. doi:10.4300/JGME-D-19-00075.1 31440327 PMC6699523

[22] Nunez-Smith M, Pilgrim N, Wynia M, Race/ethnicity and workplace discrimination: results of a national survey of physicians. J Gen Intern Med. 2009;24(11):1198-1204. doi:10.1007/s11606-009-1103-9 19727966 PMC2771235

[23] Coombs AA, King RK Workplace discrimination: experiences of practicing physicians. J Natl Med Assoc. 2005;97(4):467-477. 15868767 PMC2568696

[24] Corbie-Smith G, Frank E, Nickens HW, Elon L Prevalences and correlates of ethnic harassment in the U.S. Women Physicians’ Health Study. Acad Med. 1999;74(6):695-701. doi:10.1097/00001888-199906000-00018 10386100

[25] Fnais N, Soobiah C, Chen MH, Harassment and discrimination in medical training: a systematic review and meta-analysis. Acad Med. 2014;89(5):817-827. doi:10.1097/ACM.0000000000000200 24667512

[26] Osseo-Asare A, Balasuriya L, Huot SJ, Minority resident physicians’ views on the role of race/ethnicity in their training experiences in the workplace. JAMA Netw Open. 2018;1(5):e182723. doi:10.1001/jamanetworkopen.2018.2723 30646179 PMC6324489

[27] Wang LJ, Tanious A, Go C, Gender-based discrimination is prevalent in the integrated vascular trainee experience and serves as a predictor of burnout. J Vasc Surg. 2020;71(1):220-227. doi:10.1016/j.jvs.2019.02.064 31227409 PMC7908058

[28] McKinley SK, Wang LJ, Gartland RM, ; Massachusetts General Hospital Gender Equity Task Force “Yes, I’m the doctor”: one department’s approach to assessing and addressing gender-based discrimination in the modern medical training era. Acad Med. 2019;94(11):1691-1698. doi:10.1097/ACM.0000000000002845 31274522

[29] Kim CJ The racial triangulation of Asian Americans. Polit Soc. 1999; 27(1):105-138. doi:10.1177/0032329299027001005

[30] Association of American Medical Colleges Figure 2: U.S. physicians by Asian subgroups and sex, 2013. Accessed October 9, 2020. https://www.aamcdiversityfactsandfigures.org/section-ii-current-status-of-us-physician-workforce/index.html

[31] Devakumar D, Shannon G, Bhopal SS, Abubakar I Racism and discrimination in COVID-19 responses. Lancet. 2020;395(10231):1194. doi:10.1016/S0140-6736(20)30792-3 32246915 PMC7146645

[32] Benya FF, Widnall SE, Johnson PA, eds. Sexual Harassment of Women: Climate, Culture, and Consequences in Academic Sciences, Engineering, and Medicine. National Academies Press; 2018. 29894119

[33] Stop Street Harassment The facts behind the #metoo movement: a national study on sexual harassment and assault. Published February 21, 2018. Accessed April 24, 2020. http://www.stopstreetharassment.org/wp-content/uploads/2018/01/Full-Report-2018-National-Study-on-Sexual-Harassment-and-Assault.pdf

[34] Choo EK, van Dis J, Kass D Time’s up for medicine? only time will tell. N Engl J Med. 2018;379(17):1592-1593. doi:10.1056/NEJMp1809351 30207825

[35] Bowleg L The problem with the phrase *women and minorities*: intersectionality—an important theoretical framework for public health. Am J Public Health. 2012;102(7):1267-1273. doi:10.2105/AJPH.2012.300750 22594719 PMC3477987

[36] Crenshaw K. Mapping the margins: intersectionality, identity politics, and violence against women of color. Stanford Law Rev. 1991;43(6):1241-1299. doi:10.2307/1229039

