# Supplementary material for: Resident Physician Experiences With and Responses to Biased Patients
Source: JAMA Netw Open. 2020 Nov 23;3(11):e2021769. doi: 10.1001/jamanetworkopen.2020.21769 (PMC7684448; doi:10.1001/jamanetworkopen.2020.21769)
Supplement: Supplement. — eTable 1. Percentage of Participants Reporting Ever Experiencing Biased Patient Behavior by Race/Ethnicity and Gender eTable 2. Percentage of Participants Ever Experiencing or Witnessing Biased Patient Behavior Targeted at Specific Social Characteristics in the Last Year eTable 3. Confidence in Responding to Biased Patient Behavior eTable 4. Beliefs Around Necessity of Policies and Training on Addressing Biased Patient Behavior eTable 5. Beliefs on Adequacy of Prior Training on Biased Patient Behavior in Terms of Content and Time eAppendix. Resident Experiences With Biased Patient Behavior Survey [file jamanetwopen-e2021769-s001.pdf]

Supplementary Online Content

de Bourmont SS, Burra A, Nouri SS, et al. Resident physician experiences with and responses to biased patients. *JAMA Netw Open.* 2020;3(11):e2021769. doi:10.1001/jamanetworkopen.2020.21769

eTable 1. Percentage of Participants Reporting Ever Experiencing Biased Patient Behavior by Race/Ethnicity and Gender
eTable 2. Percentage of Participants Ever Experiencing or Witnessing Biased Patient Behavior Targeted at Specific Social Characteristics in the Last Year
eTable 3. Confidence in Responding to Biased Patient Behavior
eTable 4. Beliefs Around Necessity of Policies and Training on Addressing Biased Patient Behavior
eTable 5. Beliefs on Adequacy of Prior Training on Biased Patient Behavior in Terms of Content and Time
eAppendix. Resident Experiences With Biased Patient Behavior Survey

This supplementary material has been provided by the authors to give readers additional information about their work.

© 2020 de Bourmont SS et al. *JAMA Network Open.*

eTable 1. Percentage of Participants Reporting Ever Experiencing Biased Patient Behavior by Race/Ethnicity and Gender

**Biased Patient Behaviors**	**Latinx + Black (N=38) n (%)**	**White (N=109) n (%)**	**Asian (N=70) n (%)**	**Men (N=113) n (%)**	**Women (N=115) n (%)**
**Belittling/Demeaning Stereotypes**					
Belittling comments	34 (91.9)	77 (70.6)	61 (87.1)	68 (60.7)	113 (98.3)
Inquiries into ethnic origins	37 (97.4)	36 (33.0)	70 (100.0)	68 (60.2)	84 (73.0)
Generalizations about social identity	35 (92.1)	76 (69.7)	67 (95.7)	78 (69.0)	110 (95.7)
Confusing team members of the same race/ethnicity	28 (73.7)	70 (64.2)	69 (98.6)	83 (73.5)	93 (80.9)
Non-verbal disrespect	26 (70.3)	61 (56.0)	39 (55.7)	55 (49.1)	79 (68.7)
**Role Questioning**					
Credential/ability questioning	34 (91.9)	83 (76.2)	65 (92.9)	80 (71.4)	111 (96.5)
Assumption of non-physician status	32 (86.5)	62 (56.9)	51 (72.9)	36 (32.1)	115 (100.0)
Addressing intern or student due to social bias towards senior resident	27 (73.0)	50 (45.9)	45 (65.2)	24 (21.6)	104 (91.2)
**Sexual Harassment**	29 (78.4)	59 (54.1)	43 (61.4)	36 (32.1)	100 (87.0)
**Explicit Epithets/Rejection of Care**					
Epithets	17 (46.0)	41 (37.6)	26 (37.1)	39 (34.8)	51 (44.4)
Refusal of care	16 (43.2)	28 (25.7)	22 (31.4)	30 (26.8)	38 (33.0)
Request to change physicians	16 (43.2)	23 (21.1)	18 (26.1)	23 (20.7)	38 (33.0)

© 2020 de Bourmont SS et al. *JAMA Network Open.*

eTable 2. Percentage of Participants Ever Experiencing or Witnessing Biased Patient Behavior Targeted at Specific Social Characteristics in the Last Year

**Targeted Social Characteristics (N=230)**	**Experienced n (%)**	**Witnessed n (%)**
Ethnicity/race/national identity	118 (51.5)	205 (89.1)
Gender identity or expression	97(42.0)	169 (73.5)
Islamic faith[1]	13 (5.7)	91 (39.9)
Non-Islamic faith[1]	20 (8.8)	61 (27.0)
Sexual orientation[1]	20 (8.7)	95 (41.9)
Disability status[1]	4 (1.8)	29 (12.8)

[1]Because of missing data, N was <230 and ranged between 226-229.

© 2020 de Bourmont SS et al. *JAMA Network Open.*

eTable 3. Confidence in Responding to Biased Patient Behavior

**Confidence in Responding**	**N=231 n (%)**
**Not confident**	29 (12.6)
**Somewhat confident**	129 (55.8)
**Confident**	62 (26.8)
**Very confident**	11 (4.8)

© 2020 de Bourmont SS et al. *JAMA Network Open.*

eTable 4. Beliefs Around Necessity of Policies and Training on Addressing Biased Patient Behavior

**Necessity** (N=232)[1]	**Not necessary n (%)**	**Somewhat necessary n (%)**	**Necessary n (%)**	**Very necessary n (%)**
**Necessity of policies**	2 (0.8)	22 (9.5)	86 (37.2)	121 (52.4)
**Necessity of training for medical students**	2 (0.9)	36 (15.5)	96 (41.4)	98 (42.2)
**Necessity of training for medical residents**	1 (0.4)	25 (10.8)	97 (41.8)	109 (47.0)
**Necessity of training for medical faculty**	1 (0.4)	24 (10.4)	76 (32.9)	130 (56.3)

[1]Because of missing data, N was <233, and ranged between 231-232.

© 2020 de Bourmont SS et al. *JAMA Network Open.*

eTable 5. Beliefs on Adequacy of Prior Training on Biased Patient Behavior in Terms of Content and Time

**Adequacy**	**Content (N=203) n (%)**	**Time (N=232) n (%)**
**Inadequate**	59 (29.1)	91 (39.2)
**Somewhat adequate**	91 (44.8)	80 (34.5)
**Adequate**	44 (21.7)	52 (22.4)
**More than adequate**	9 (4.4)	9 (3.9)

© 2020 de Bourmont SS et al. *JAMA Network Open.*

eAppendix. Resident Experiences With Biased Patient Behavior Survey

---

Start of Block: Consent document

Q1.1 **UNIVERSITY OF CALIFORNIA, SAN FRANCISCO**
CONSENT TO PARTICIPATE IN A RESEARCH STUDY
Study Title: When Patients Demean Clinicians Because of Social Bias We are asking you to consider taking part in a research study conducted by Shalila de Bourmont, Arun Burra, and Alicia Fernandez, MD in the UCSF Department of Medicine, Neveen El-Farra, MD and Jodi Friedman, MD in the UCLA Department of Medicine, and Dinushika Mohottige, MD, Caroline Sloan, MD, Daniella Zipkin, MD, and Aimee Zaas, MD in the Duke Department of Medicine. The purpose of this study is to determine medical residents' perceptions of encounters with patients who exhibit demeaning behaviors toward their clinicians because of social biases. We aim to determine the range and prevalence of demeaning patient behavior, what resources and approaches residents use in response to such events, and the factors that prevent or facilitate responses. You are being asked to participate in this study because you are a 2nd or 3rd year internal medicine resident at UCSF, UCLA, or Duke. About 230 people will take part in this study. If you agree to be in the study, you will fill out a short survey that includes open-ended questions. **The survey should take about 8 minutes or less to complete.** It is possible that some of the survey questions may make you feel uncomfortable. You may choose not to answer any questions that you wish or decline to take the survey altogether. CONFIDENTIALITY: **The survey is anonymous.** Your survey answers will initially be stored with Qualtrics in a password protected electronic format. Data will later be downloaded and stored on UCSF MyResearch, a secure data hosting service. No individual identities or other personal information will be collected or used in any reports or publications that may result from this study. **All participants will be eligible for a ten-dollar gift card**. Receipt of the incentive is optional. If you choose to obtain the gift card, you will be directed to a form where you will provide your institutional email address. The gift card will then be emailed to you. Although the student-researchers will have access to your email address, your email address cannot be linked to your survey responses. This will ensure confidentiality. The information in this study may help provide insight into the experiences of residents that have witnessed or experienced socially biased, demeaning behavior from patients. This information will also guide the development of policies and protocols on responding to biased patients for use locally and more broadly that balance respect for patient autonomy and healthcare providers' rights. There are no costs to you as a result of taking part in this study. If you have any questions about the study, you may email Arun Burra at Arun.Burra@ucsf.edu, Shalila de Bourmont at Shalila.DeBourmont@ucsf.edu, or Dr. Fernandez at Alicia.Fernandez@ucsf.edu. If you wish to ask questions about the study or your rights as a research participant to someone other than the researchers, or if you wish to voice any problems or concerns you may have about the study, please call the UCSF Institutional Review Board at (415) 476-1814.

PARTICIPATION IN RESEARCH IS VOLUNTARY. **You are free to decline to be in this study, or to withdraw from it at any point without penalty to you in any way.** RESOURCES WITHIN THE RESIDENCY: For support within the UCLA residency for any of the issues addressed in the survey, please reach out to Dr. Neveen El-Farra (nelfarra@mednet.ucla.edu) or any

member of residency leadership.   CONSENT: **If you wish to participate in this study, please begin the survey.**

End of Block: Consent document

Start of Block: Typology_prevalence

Q2.1 **We are interested in residents' perceptions of encounters with biased patients (i.e. patients who exhibit demeaning behaviors toward physicians because of social biases such as**

© 2020 de Bourmont SS et al. *JAMA Network Open.*

racism). Biased behavior includes actions or speech that rejects, degrades, or belittles physicians.

Q2.2 In the last year, how often did YOU DIRECTLY EXPERIENCE biased patient behavior toward YOU characterized by:

© 2020 de Bourmont SS et al. *JAMA Network Open.*

	Never (1)	Once or twice per year (2)	A few times per year (3)	About once per month (4)	More than once per month (5)	Once per week or more (6)
Comments or compliments that belittle clinicians (e.g., calling women doctors “honey” instead of “doctor”) (1)	○	○	○	○	○	○
Assertive inquiries into ethnic origins (e.g., "where are you really from?") (2)	○	○	○	○	○	○
Generalizations about other people who share your ethnicity, gender, sexual orientation, or other social characteristics (3)	○	○	○	○	○	○
Confusing you with others of the same ethnicity/race (4)	○	○	○	○	○	○
Non-verbal disrespect (e.g., disrespectful gestures or a tattoo of a swastika) (5)	○	○	○	○	○	○
Questioning credentials/ability because of social bias - not because of trainee status (e.g., “you don’t look like a doctor”) (6)	○	○	○	○	○	○

© 2020 de Bourmont SS et al. *JAMA Network Open.*

Assumptions that you are ancillary staff/a non-physician because of social bias (7)	○	○	○	○	○	○
Patient addresses student or intern despite you being the senior doctor present because of social bias (8)	○	○	○	○	○	○
Overt verbal or non-verbal sexual advances/sexual harassment (9)	○	○	○	○	○	○
Epithets/hate speech/slurs/name calling (e.g., racial slurs, gender slurs, etc.) (10)	○	○	○	○	○	○
Explicit refusal/rejection of care because of social bias (11)	○	○	○	○	○	○
Explicit request to change to another physician because of social bias (12)	○	○	○	○	○	○
Other (please elaborate): (13)	○	○	○	○	○	○

End of Block: Typology_prevalence

Start of Block: Target_experience

© 2020 de Bourmont SS et al. *JAMA Network Open.*

Q3.1 In the last year, how often did you DIRECTLY EXPERIENCE biased patient behavior based on the following characteristics (whether the patient presumed the characteristic correctly or not):

	Never (1)	Once or twice per year (2)	A few times per year (3)	About once per month (4)	More than once per month (5)	Once per week or more (6)
Ethnicity/race/national identity (1)	○	○	○	○	○	○
Gender identity or expression (2)	○	○	○	○	○	○
Muslim faith (3)	○	○	○	○	○	○
Non-Muslim faith (7)	○	○	○	○	○	○
Sexual orientation (4)	○	○	○	○	○	○
Disability status (5)	○	○	○	○	○	○
Other (please elaborate) (6)	○	○	○	○	○	○

End of Block: Target_experience

Start of Block: Target_witness

© 2020 de Bourmont SS et al. *JAMA Network Open.*

Q4.1 In the last year, how often did you DIRECTLY WITNESS biased patient behavior towards another physician based on the following characteristics (whether the patient presumed the characteristic correctly or not):

	Never (1)	Once or twice per year (2)	A few times per year (3)	About once per month (4)	More than once per month (5)	Once per week or more (6)
Ethnicity/race/national identity (1)	○	○	○	○	○	○
Gender identity or expression (2)	○	○	○	○	○	○
Muslim faith (3)	○	○	○	○	○	○
Non-Muslim faith (7)	○	○	○	○	○	○
Sexual orientation (4)	○	○	○	○	○	○
Disability status (5)	○	○	○	○	○	○
Other (please elaborate) (6)	○	○	○	○	○	○

End of Block: Target_witness

Start of Block: Open ended_typology

Q5.1 Open-ended: Please tell us about a specific incident, what impact it had on you, and how it was addressed.

____

____

____

____

____

End of Block: Open ended_typology

© 2020 de Bourmont SS et al. *JAMA Network Open.*

Start of Block: Branch point (skip questions 5-7)

Q6.1 If you have NEVER experienced or witnessed biased patient behavior during your time in residency, please select "I have NEVER experienced or witnessed biased patient behavior." Otherwise, please select "continue survey." **Please note you will not be able to edit your prior responses after this point.**

○ I have NEVER experienced or witnessed biased patient behavior (1)

○ Continue survey (2)

End of Block: Branch point (skip questions 9.1 and 10.1 if have never experienced or witnessed biased patient behavior, otherwise continue to question 7.1)

Start of Block: Block_training

Q7.1 How much training have you received on dealing with biased patient behavior in:

	None (1)	<1 hr (2)	1-2 hrs (3)	>2hrs (4)
Medical School (1)	○	○	○	○
Residency (2)	○	○	○	○

Skip To: Q7.3 If How much training have you received on dealing with biased patient behavior in: [None] (Count) = 2

Q7.2 How would you rate the adequacy of your prior training on biased patient behavior in terms of CONTENT?

○ Inadequate (1)

○ Somewhat adequate (3)

○ Adequate (4)

○ More than adequate (5)

© 2020 de Bourmont SS et al. *JAMA Network Open.*

Q7.3 How would you rate the adequacy of your prior training on biased patient behavior in terms of TIME?

- ○ Inadequate (1)
- ○ Somewhat adequate (3)
- ○ Adequate (4)
- ○ More than adequate (5)

Q7.4 How necessary do you think training on dealing with biased patient behavior is for:

	Not necessary (1)	Somewhat necessary (2)	Necessary (3)	Very necessary (4)
Medical students (1)	○	○	○	○
Residents (2)	○	○	○	○
Faculty (3)	○	○	○	○

Q7.5 Open-ended: Please specify any content you would want included in such a training.

____

____

____

____

____

© 2020 de Bourmont SS et al. *JAMA Network Open.*

Q7.6 How confident are you in your ability to respond to biased patient behavior in your workplace?

- Not confident (1)
- Somewhat confident (2)
- Confident (3)
- Very confident (4)

Q7.7 How necessary do you think institutional policies are for addressing biased behavior from patients?

- Not necessary (1)
- Somewhat necessary (2)
- Necessary (3)
- Very necessary (4)

Q7.8 Open-ended: What specific institutional policies, if any, do you think would be helpful in addressing biased patient behavior?

____

____

____

____

____

End of Block: Block_training

Start of Block: Block_Demographics

Q8.1 **Please provide the following demographic information:**

© 2020 de Bourmont SS et al. *JAMA Network Open.*

Q8.2 Year of residency

- 2 (1)
- 3 (2)

Q8.3 Institution where you are a resident

- Duke (3)
- UCLA (1)
- UCSF (2)

Q8.4 Race/ethnicity (you may choose more than one)

- White (1)
- Black or African American (2)
- Hispanic or Latinx (6)
- Asian (4)
- Native Hawaiian or Pacific Islander (5)
- Native American or Alaska Native (3)
- Other (7) ____

© 2020 de Bourmont SS et al. *JAMA Network Open.*

Q8.5 Gender

- ○ Male (1)
- ○ Female (2)
- ○ A gender not listed here (3)
- ○ Prefer not to say (4)

Q8.6 Do you identify as being gay, lesbian, bisexual, transgender, queer (LGBTQ), or as another sexual or gender minority?

- ○ Yes (1)
- ○ No (2)

Q8.7 Are you an immigrant to the United States?

- ○ Yes (1)
- ○ No (2)

End of Block: Block_Demographics

Start of Block: Block_response

© 2020 de Bourmont SS et al. *JAMA Network Open.*

Q9.1 Thinking about these incidents as a group, how often did you or your team utilize one of the following responses?

	Never (1)	Sometimes (2)	About half of the time (3)	Most of the time (4)	Always (5)
Responded on my own to set limits with patient (1)	o	o	o	o	o
Debriefed with non-team member (e.g., friends or family) (9)	o	o	o	o	o
Debriefed with a member of the team (7)	o	o	o	o	o
Created a team response plan (2)	o	o	o	o	o
Reported demeaning behavior/language to my attending or chief resident (3)	o	o	o	o	o
Reported demeaning behavior/language to my institution (8)	o	o	o	o	o
Switched patient to another team member (4)	o	o	o	o	o
Did not address incident (5)	o	o	o	o	o
Other (please describe the response and the context in which it was used): (6)	o	o	o	o	o

End of Block: Block_response

Start of Block: Block_barriers

Q10.1 To what degree have the following factors negatively impacted your ability to address biased patient behavior?

© 2020 de Bourmont SS et al. *JAMA Network Open.*

	No impact (1)	Minimal impact (2)	Some impact (3)	Significant impact (4)
Prioritizing the clinical care of the patient (1)	○	○	○	○
Feeling unsupported by the team, seniors, or institution (2)	○	○	○	○
Lack of knowledge or skills about how to properly respond (3)	○	○	○	○
Perceived ineffectiveness of responding (4)	○	○	○	○
Feeling emotionally overwhelmed (5)	○	○	○	○
Other (please elaborate) (6)	○	○	○	○

End of Block: Block_barriers

© 2020 de Bourmont SS et al. *JAMA Network Open.*
